# Advancing Mediterranean food integrity: assessing a digital tool for consumer empowerment and traceability

**DOI:** 10.1007/s13197-025-06230-1

**Published:** 2025-02-18

**Authors:** Gloria Cea, Ivana Lombroni, Beatriz Merino-Barbancho, Adrián Dantas, Irene Mallo, Nikos Tsotsolas, Georgios F. Banias, María Teresa Arredondo

**Affiliations:** 1 Life Supporting Technologies– LifeSTech, Universidad Politécnica de Madrid, Madrid, Spain; 2 Green Projects SA, R&D Department, Chalandri, Attica, Greece; 3 Institute for Bio-Economy and Agri-Technology (iBO), Centre for Research and Technology—Hellas (CERTH), Thermi, Greece

**Keywords:** Food transparency, Food traceability, Mediterranean food products, Consumer, Digital solution, Living lab

## Abstract

The Mediterranean Diet is widely renowned for its benefits in promoting longevity and well-being. However, ensuring the authenticity and traceability of Mediterranean food products remains a significant challenge. To tackle this, the food industry must adopt transparent and reliable supply chain practices that includes leveraging innovative technologies, essential to meet consumer demands, enhance efficiency, and promote sustainability. This study aims to validate the feasibility of the Med Food TTHubs Consumer App ensuring that the digital solution meets the needs and expectations of the target audience facilitating consumer adoption. User perceptions were assessed using the System Usability Scale (SUS) and the Technology Acceptance Model (TAM) with 21 participants. SUS analysis showed an average score of 74.5, indicating the solution’s acceptability fell short of good usability criteria. Notably, 66.7% found it acceptable (SUS Score ≥ 68), and 47.6% rated it as having good usability (SUS Score ≥ 80). TAM revealed a positive perception, showing potential acceptance and adoption. The acceptable Cronbach’s Alpha value of 0.721 indicates internal consistency. This digital solution emerges as an encouraging tool to enhance transparency, traceability, and consumer empowerment in the food sector. Despite identified usability issues, its user-friendly interface and comprehensive information contribute to informed decision-making and healthier food choices.

## Research highlights

- Study participants reveals a favourable attitude towards adopting new digital solutions for food.
- Positive disposition suggests technology readiness for food info accessibility and traceability.
- Consumer App is acceptable but it lacks a seamless interaction.
- Barriers hinder a widespread adoption of this digital solution despite its potential benefits.
- Enhance the Consumer App for better usability, accessibility and overall user experience.

## Introduction

The Mediterranean Diet is a healthy and sustainable way of eating that promotes longevity and overall health recognized as one of the healthiest diets in the world (Guasch-Ferré and Willett 2021). It is based on fresh and flavourful ingredients such as vegetables, fruits, legumes, nuts, whole grains, fish, and olive oil. These ingredients are rich in nutrients and antioxidants that provide numerous health benefits. It provides a balance of macronutrients and micronutrients, rich in fibre, healthy fats, vitamins, and minerals; it is low in processed foods, refined sugars, and unhealthy fats (Mazzocchi et al. 2019) focused on natural, whole foods that are minimally processed.

The lack of trust in the origin and quality of raw materials in Mediterranean food products is a major issue that poses obstacles to the sustainability and competitiveness of small and medium agro-enterprises in both local and global value chains. Even though these products are recognized for their high nutritional and sensorial value, consumers are increasingly demanding high-quality food products with proven origin and documented quality. To meet these demands, the food industry must adapt and create a more transparent and trustworthy supply chain. By intensively monitoring and tracing the supply chain, better product and information management can be achieved, resulting in reduced waste and improved efficiency.

The challenge pertaining to the authenticity and traceability of food products lies in the difficulty of guaranteeing the veracity of claims and the adherence to safe and ethical production practices. Various factors contribute to this complex issue, including the widespread globalization of the food supply chain, the prevalence of counterfeiting and fraudulent activities, concerns surrounding food safety, environmental considerations, and ethical dilemmas. To address these problems, efforts are underway to improve the authenticity and traceability of food products, such as the use of innovative technologies to track food products from farm to fork and the development of new testing methods to detect food fraud. However, these efforts are still in the early stages and much work remains to be done to ensure the safety, authenticity, and ethical production of the food we eat.

The industry should move towards this integrated whole-chain traceability process while balancing regulatory requirements and implementing proven supply chain management business practises. As consumer demands, public health concerns, and business needs converge, the transparency and sustainability of the food supply chain must be improved.

This paper presents the study that evaluates the feasibility and likelihood of success of the Med Food TTHubs Consumer App, a digital solution that enables consumers to access comprehensive information about food products, from farm to fork. The study aims to ascertain the tangible advantages that the digital solution would offer to both consumers and producers in the agri-food industry and the results will assist in the decision-making process regarding whether to proceed with the market launch of the solution. An assessment was conducted on the potential effectiveness of utilizing the Consumer App to achieve the following: enhancing transparency and traceability by presenting consumers with comprehensive details regarding the origin, authenticity, traceability, nutrition, and quality of food products; improving the consumer experience through convenient access to information such as nutritional facts and tips about food products; fostering consumer trust by facilitating the sharing of information through an e-platform, thereby establishing a stronger connection across the agri-food supply chain; evaluating the potential market demand for the app, identifying the target audience, and understanding their preferences and requirements concerning food product information. This feasibility study did not involve the use of controls, randomization, or any specific interventions.

## Materials and methods

### Med food TT hubs

Med Food TTHubs is a PRIMA project (European Union’s Horizon 2020 Research and Innovation programme, s. f.) that aims to establish comprehensive tracing practices throughout the entire distribution channel of Mediterranean food products. By implementing full-path tracing from seed to shelf, the project seeks to achieve safer and more sustainable food products. It utilizes state-of-the-art technologies, such as blockchain, or Internet of Things (IoT) solutions, to ensure transparency and authenticity in the food supply sector, including proofs of authenticity for final products and ingredients, as well as detailed nutritional information. The project’s core concept revolves around the establishment of seven Trace & Trust Hubs (TTHubs), which serve as collaborative platforms connecting various stakeholders and fostering transparency and trust throughout the supply chain.

It provides a solution that aims to create safer and more sustainable Mediterranean food products while providing full transparency concerning the traceability and authenticity of these products. This helps to create an end-to-end trust-chain in the food sector, which is critical for meeting the demands of consumers and ensuring the sustainability and competitiveness of SMEs in the global marketplace. The project aims to create a more transparent and traceable supply chain for Mediterranean food products allowing consumers to track the journey of their food products from cultivation and breeding to packaging and transport, ensuring that they are of high quality and have a proven origin.

#### Consumer app

The Consumer App is a mobile application developed to facilitate the exploration and access to comprehensive information about Mediterranean food products built on top of the Med Food TT Hubs e-platform. Through its user-friendly interface, the app enables users to retrieve detailed data regarding each product, encompassing aspects such as origin, nutritional composition, and certifications, thereby empowering consumers to make informed decisions.

One of the prominent features of the app revolves around its emphasis on enhancing transparency and traceability within the food supply chain. By allowing users to scan product QR codes or Bluetooth Beacons, the app grants them access to intricate insights into the entire journey of the respective product, spanning from its production methods, quality control measures, to the obtained certifications. It enables consumers to make conscious choices and actively support sustainable and ethical food practices.

The overarching goal of the Med Food TT Hubs Consumer App is to provide a seamless and enjoyable experience for individuals keen on exploring and embracing the Mediterranean diet and lifestyle. By capitalizing on technological advancements, the app not only promotes healthy eating habits but also facilitates the establishment of connections between consumers and local producers, acting as a catalyst for fostering sustainability within the food industry.

### Study settings: i·Apetito and LifeSpace

The study was conducted at i·Apetito facilities (LifeSTech– UPM, s. f.), located in Madrid, Spain. i·Apetito is the Trace & Trust HUB dedicated to promoting food sovereignty and nutrition for a healthy lifestyle and well-being. It is part of the LifeSpace (LifeSTech - UPM, s. f.) infrastructure, that operates as a specialized Living Lab dedicated to the advancement of technologies promoting health and well-being. It is an esteemed member of the European Network of Living Labs– ENOLL (ENOLL, s. f.) and the Madrid Research Infrastructures Network, showcasing its commitment to research and innovation. A set of cutting-edge technological components comprise a configurable and all-encompassing infrastructure, facilitating the seamless deployment and evaluation of diverse applications. This versatile framework fosters rapid prototyping and early user involvement, ensuring the efficient development and testing of various technological solutions. Its mission centers around collaborative efforts to co-create and cultivate technology-driven services that contribute to the prevention, care, and enhancement of individuals’ health and well-being. Moreover, the lab actively supports social inclusion initiatives and endeavours to empower and enable independent living among vulnerable and dependent populations. Through close collaboration with a diverse range of users, LifeSpace seeks to engender technological advancements that address the specific needs and requirements of these user groups, ultimately promoting their overall quality of life and well-being.

Simulating a food product market will provide insights into customer behaviour that may not be easy to obtain through other means. Indicatively, if the validation process uncovers customer resistance towards the app’s current functionality or identifies weaknesses, developers can adjust the solution accordingly. This enhances the likelihood of creating a successful product that satisfies its target audience and facilitates the creation of a controlled environment, wherein it is possible to test and validate the Consumer App under conditions that simulate real-world settings.

**Fig. 1:**
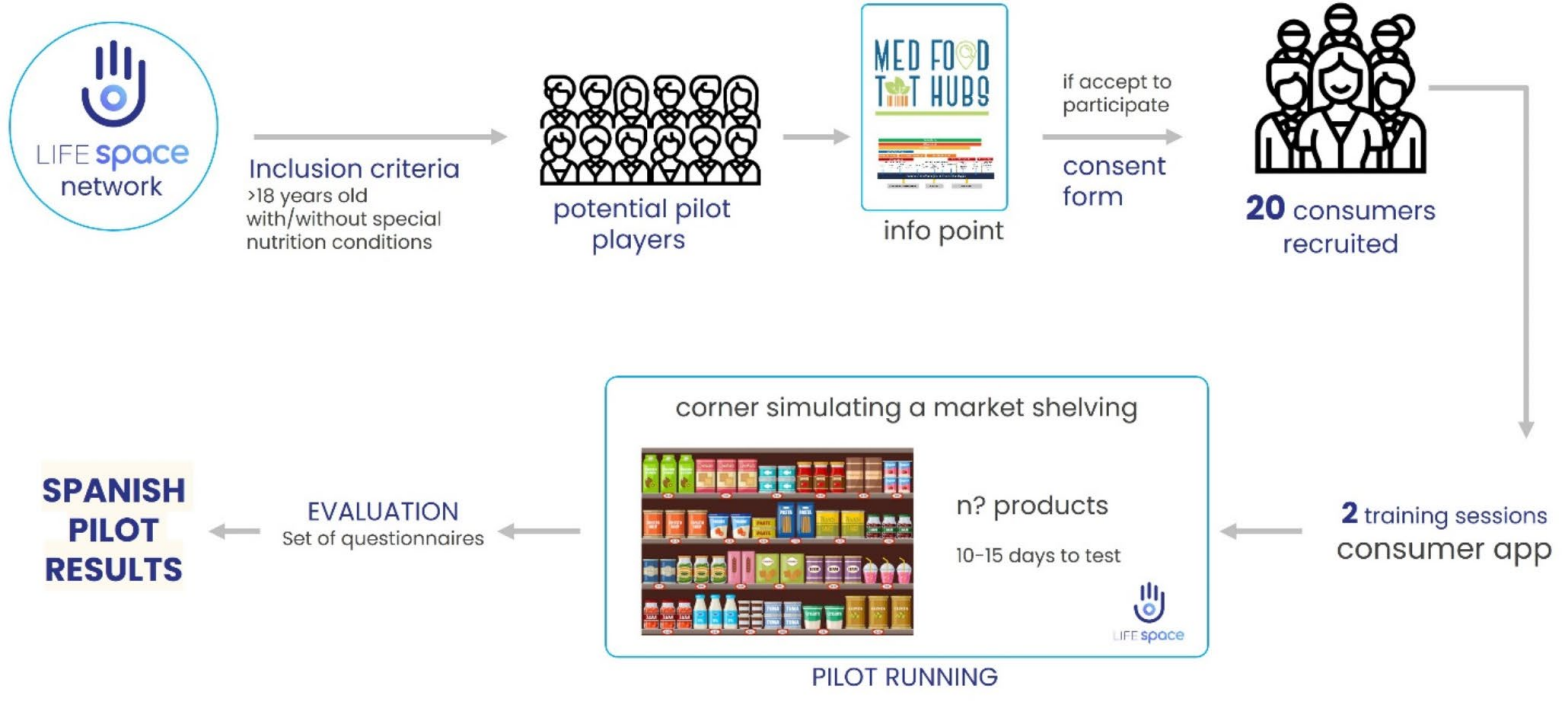
Methodology outline

Individuals of legal age, irrespective of the presence or absence of specific dietary requirements such as coeliac disease, food intolerances, food allergies, were contacted through the LifeSpace network with an invitation to participate. Upon voluntary agreement, pilot participants were obliged to provide their informed consent by signing a consent form. Subsequently, comprehensive training sessions were administered to ensure participants’ familiarity with the study’s objectives and the operational functionalities of the Consumer app. Throughout the study duration, rigorous monitoring of participants’ app engagement was conducted by the researchers, thereby facilitating data collection and feedback acquisition for subsequent analysis and evaluation (Fig. 1). This enables the validation of the digital solution in a more realistic environment, facilitating the identification of any issues that may not appear during development or lab testing. Consequently, we could obtain direct feedback and first impressions from users that can be leveraged to refine and enhance the solution to better meet user needs.

### Evaluation instruments

#### Usability

The System Usability Scale (SUS) questionnaire is a Likert scale for measuring the perceived usability of a system or product. It was developed by John Brooke in 1986 (Brooke 1986) and consists of ten statements that users rate on a 5-point Likert scale, ranging from “Strongly Agree” to “Strongly Disagree” and is designed to gather user feedback on factors such as complexity, consistency, and ease of use.

The SUS questionnaire serves as instrument to evaluate user perceptions of system usability swiftly and precisely. Although the original SUS questionnaire was designed as a one-dimensional “quick and dirty strategy to assess usability, research conducted in the past 25 years has uncovered insightful dimensions to contextualize and contrast individual SUS scores.

To offer a comprehensive evaluation of the usability of the Consumer App, an open-source web-based Analysis Toolkit (Blattgerste et al. 2022) were utilized for the SUS questionnaire. This toolkit enables to calculate SUS measurements, analyse them based on previous research’s suggested insights and contextualization scales, and generate graphical representations of the data.

#### Technology acceptance

The tool selected is the Technology Acceptance Model (TAM) questionnaire, which is a widely used tool for predicting the users’ acceptance and usage of new technology. It was originally proposed by Davis in 1989 (Davis et al. 1989), and has since been modified and extended by many researchers (Nair and Das 2011; Tarhini et al. 2015; Teo 2011). The original objective of this assessment was to quantify likelihood of technology acceptance.

The TAM questionnaire typically comprises items that pertain to users’ attitudes toward the technology, their perceived usefulness of the technology, and their perceived ease of use of the technology. Participants are usually asked to rate their level of agreement or disagreement with each statement on a scale, such as a 5-point Likert scale ranging from “Strongly Disagree” to “Strongly Agree”. The responses can then be analysed to determine the degree to which users accept the new technology based on their attitudes, perceived usefulness, and perceived ease of use.

#### Open-ended questions

At the conclusion of the survey, we have included three optional open-ended questions to gain a deeper understanding of our pilot players, who are consumers, and their requirements.

- *Q1. Could you let us know if you think this solution is useful? In what sense? What advantages do you see and what disadvantages?*
- *Q2. Do you find any barriers*, *whether technological*, *social or personal when using this solution? Which ones? Why?*
- *Q3. In a sentence*, *what would you say is the value you take away from this solution?*

The objective is to obtain context behind their actions, investigate the reasons for their satisfaction and dissatisfaction with the Consumer App, and develop insights into their needs. Open-ended questions necessitate elaboration from respondents, and they cannot be answered with a simple ‘yes’ or ‘no’. This feature enables to gather honest feedback, as participants can describe their experiences in their own words. From the evaluator’s perspective, these questions provide an opportunity to empathize with the audience (consumers). The results were analysed using spreadsheets to view qualitative trends and to spot significant elements with word cloud visualizations.

## Results

The success of a new solution is largely dependent on its ability to meet the expectations and requirements of its intended users. To ensure this, a feasibility study was conducted, which is considered an essential tool for determining the viability of a new idea and provides valuable insights into how to improve the product prior to its launch. This section presents the results of the feasibility study conducted on the Consumer App, with a focus on assessing its potential success based on various factors, such as usability, technology acceptance, usefulness, value, and barriers. By examining the study’s findings, we aim to elucidate the key factors that can influence the success or failure of this digital solution and provide valuable insights to enhance the app’s chances of success.

### Participants profile

Table 1 presents the demographic characteristics of the 21 study participants. On average, the participants were 30 years old (SD 6), with the majority being male (12/21, 57.14%) and possessing a Master’s degree or higher (18/21, 85.72%). Twenty out of the twenty-one participants (95.24%) had advanced or excellent technological knowledge. The majority of participants reported having no dietary restrictions (18/21, 85.72%), while one participant had multiple food allergies or intolerances (1/21, 4.76%).

**Table 1:** Participants characteristics

	Mean	SD
Age	30,38	6,16
**Characteristics**	**N**	**%**
**Gender, n (%)**		
Female	9	42,86%
Male	12	57,14%
Transgender	0	0%
Non-binary/non-conforming	0	0%
Prefer not to respond	0	0%
**Education, n (%)**		
ISCED 0: Early childhood education	0	0%
ISCED 1: Primary education	0	0%
ISCED 2: Lower secondary education	1	4,76%
ISCED 3: Upper secondary education	0	0%
ISCED 4: Post-secondary non-tertiary education	0	0%
ISCED 5: Short-cycle tertiary education	0	0%
ISCED 6: Bachelor’s or equivalent level	2	9,52%
ISCED 7: Master’s or equivalent level	12	57,15%
ISCED 8: Doctoral or equivalent level	6	28,57%
**Technology knowledge, n(%)**		
Basic	0	0%
Intermediate	1	4,76%
Advanced	12	57,14%
Excellent	8	38,10%
**Dietary restrictions, n(%)**		
Lactose intolerance	1	4,76%
Fruits	2	9,52%
Nuts	1	4,76%
None	18	85,72%

### Usability

The usability of the Consumer App is determined based on the SUS questionnaire. To calculate the SUS score, the responses to statements 1, 3, 5, 7, and 9 are summed and multiplied by 2.5. The responses to statements 2, 4, 6, 8, and 10 are subtracted from 5 and then summed, and the result is also multiplied by 2.5. The two scores are added together to get the final SUS score, which ranges from 0 to 100. Higher scores indicate higher usability, with a score of 68 considered average and a score above 80 indicatives of good usability.

**Fig. 2:**
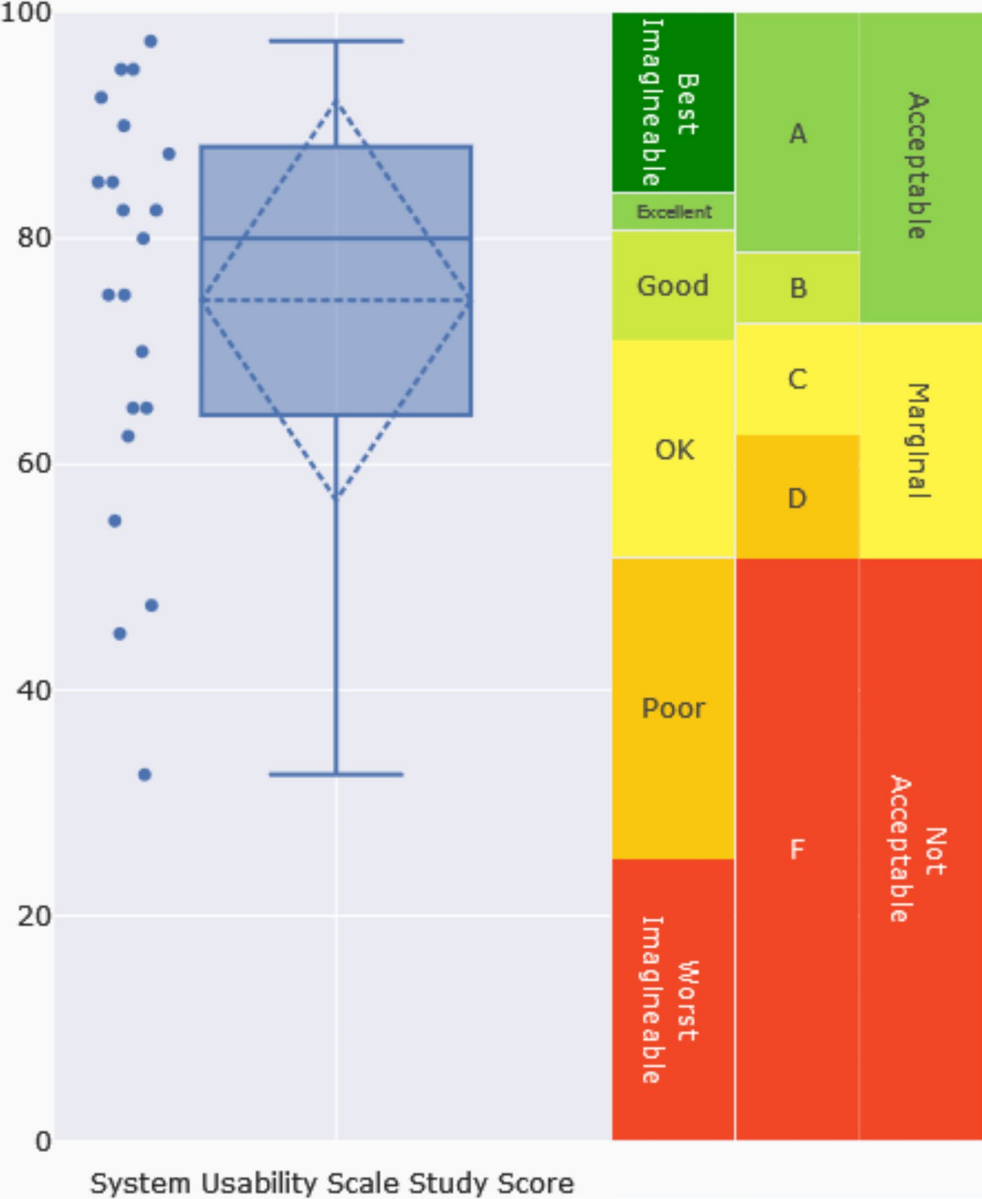
SUS Study Score

Figure 2 represents the distribution of the final SUS Score per participant provided by the SUS Analysis Toolkit. On the right side, the different scales: 1) the ***acceptability scale*** that contextualizes SUS study scores on descriptions ranging from “Not Acceptable” over “Marginally acceptable” to “Acceptable”. This scale is based on data from (Bangor et al. 2008) and derived from implications of the grading and ***adjective scales*** ranging from “Worst Imaginable”, “Poor”, “Ok”, “Good”, “Excellent”, “Best Imaginable”, where each of the 1-100 SUS scores corresponds to one of these adjectives.

Upon analysing the different questions, the score and interpretation reveal that the mean SUS score is 74.5. Consequently, the Consumer App is considered **Acceptable** according to the acceptability scale and **Good** according to the adjective scale. However, it cannot be classified as a technological solution with good usability. The analysis further indicates that 66.7% of users rated the Consumer App as acceptable in terms of usability (SUS Score ≥ 68), while 47.6% regarded it as having good usability (SUS Score ≥ 80).

**SUS Study Score = 74**,**52 (SD = 17**,**69; Median = 80)**.

**Fig. 3:**
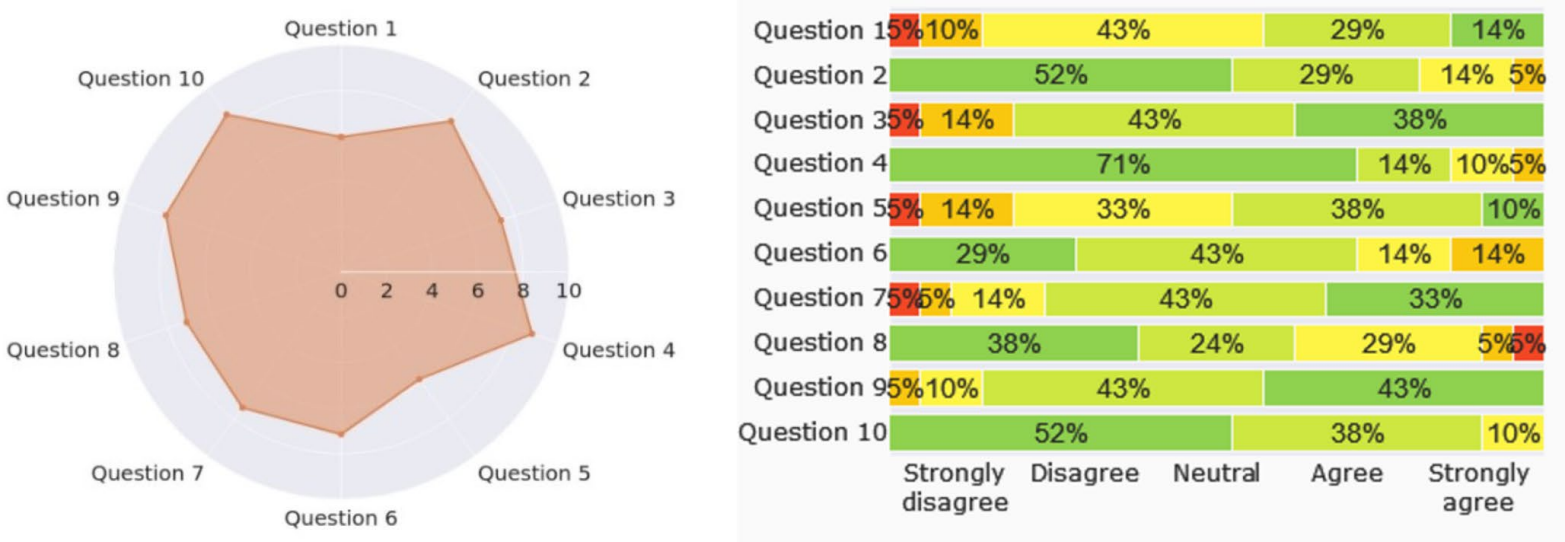
SUS per question scores

The per-item values (Fig. 3) are normalized values between 0 and 10, which represent their contribution to the SUS study score and not the Likert scale values in the questionnaire where even-numbered questions are formulated negatively.

The individual questions’ contribution towards the SUS study score is the following:

**Table:** 

• Question 1: 5.95	• Question 6: 7.14
• Question 2: 8.21	• Question 7: 7.38
• Question 3: 7.38	• Question 8: 7.14
• Question 4: 8.81	• Question 9: 8.1
• Question 5: 5.83	• Question 10: 8.57

The conclusiveness is based on data from Tullis et al. (Tullis and Stetson 2004), where the authors endeavoured to identify the necessary sample sizes for different usability questionnaires to achieve conclusiveness. The findings revealed that with a sample size of eight, a SUS study is already 75% conclusive, with 10 participants, 80%, and with 12 or more, 100%. In this study, the number of subjects is 21, resulting in a **Conclusiveness of 100%**.

Based on the results of this analysis, it can be stated that the study is conclusive, and the rating of the Consumer App is deemed acceptable, but its usability is not considered good. As such, there is a need to improve the app in several areas, which will be identified in the remaining evaluation tools.

### Technology acceptance

In this study, the TAM was used to evaluate the consumers’ adoption of a new digital solution that enables access to comprehensive information about food products along the whole supply chain including origin, authenticity, traceability and nutritional values. It is based on four parameters: ease of use, usefulness, attitude, and intention to use. Once the TAM questionnaire is administered and the responses are collected, the results are analysed using statistical techniques to gain insights into the acceptance and usage of the technology being studied, i.e. Med Food TT Hubs Consumer App.

The results of the TAM questionnaire provide insights into the acceptance and usage of a technology and can help inform decisions about its development, marketing, and implementation. The TAM was returned 21/21 participants and results indicate the Consumer App to be acceptable (Table 2).

**Table 2:** TAM number of responses

	Strongly Disagree	Disagree	Neutral	Agree	Strongly Agree
**Usefulness (n)**					
The use of the application could help me to execute my activities more rapidly	1	3	10	6	1
The use of the application could help me	1	0	5	10	5
The use of the application may imply major changes in my current way of doing	1	3	8	5	4
**Ease of use (n)**					
I think that I could easily learn how to use the application	0	3	1	6	11
I think it is a good idea to use the application to get specific info on a food product (description, tips, events, nutritional)	0	0	1	7	13
I think that my work center has the necessary infrastructure to support my use of the application	0	1	6	8	6
**Intention to Use (n)**					
I have the intention to use the application when it becomes available	1	3	9	4	4
**Attitude (n)**					
I feel comfortable with information and communication technologies	0	0	0	4	17

#### Descriptive statistics of the theoretical variables

The analysis of the responses to the TAM questionnaire revealed that the participants had an overall positive perception of the technology under consideration (Table 3). The highest-rated parameter was *Attitude*, with a score of 4.81, indicating that the respondents held a favourable attitude towards the technology. This is a crucial finding as it suggests that the respondents are likely to accept and adopt the technology. The second-highest rated parameter was *Ease of Use*, with a score of 4.22, signifying that the technology was perceived as easy to use by the respondents.

**Table 3:** TAM descriptive statistics

	Easy of Use	Usefulness	Attitude	Intention to Use
Mean	4.22	3.46	4.81	3.33
SD	0.90	1.02	0.39	1.08
Correlation with IU	0.53	0.78	0.04	1.00

The parameters of *Usefulness* and *Intention to Use* had similar scores, with a rating of 3.46 and 3.33, respectively. These results suggest that while the respondents acknowledged the usefulness of the technology, they were somewhat hesitant in their intention to use it. This finding may be attributed to various factors, such as lack of awareness, familiarity with the technology, or compatibility issues.

It is worth noting that the positive ratings of attitude and ease of use are promising indicators for the technology’s success. Furthermore, the findings also highlight the need to address any concerns or uncertainties regarding the usefulness and intention to use the technology. By addressing these issues, designers and developers can enhance the technology’s usability and increase the likelihood of its successful adoption.

#### Correlation analysis

To determine the factors that are predictive of technology adoption, a correlation analysis was conducted to evaluate the relationship between the perceived ease of use, usefulness, attitude, and intention to use the Consumer App. The analysis provides valuable insights into the factors that can influence the adoption of the technology. The results of the correlation analysis revealed a strong positive correlation between usefulness and intention to use, with a correlation coefficient of 0.78. This indicates that the perceived usefulness of the Consumer App has a significant influence on the intention to use it. This finding suggests that users are more likely to adopt the technology if they perceive it as useful in meeting their needs.

Furthermore, the analysis revealed a moderate positive correlation between ease of use and intention to use, with a correlation coefficient of 0.53. This indicates that users are more likely to adopt the technology if they perceive it as easy to use. A technology that is easy to use can reduce the user’s perceived effort and increase the likelihood of its successful adoption.

In contrast, the analysis found a lower positive correlation between attitude and intention to use, with a correlation coefficient of 0.04. This indicates that attitude may have a weaker influence on the intention to use the technology. Attitude may be shaped by various factors, such as personal preferences, prior experience with similar technologies, or social norms. However, it may not necessarily translate into a strong intention to use the technology.

#### Reliability

The reliability of the questionnaire has been assessed by calculating its internal consistency using Cronbach’s alpha (Bland and Altman 1997). Cronbach’s alpha is a measure of internal consistency reliability used to assess the reliability, or consistency, of a scale or measure composed of multiple items. It is a statistical measure that indicates how closely related a set of items are as a group, and it reflects the extent to which the items measure the same underlying construct or dimension.

**Cronbach’s Alpha = 0.721**

Since we calculated Cronbach’s Alpha to be 0.721, we would say that the internal consistency of this questionnaire is “Acceptable.”

### Qualitative results

#### Usefulness

The feedback collected underscores the significance of the Consumer App in empowering users to make informed decisions regarding their food choices, promoting healthy dietary habits, and instilling consumer trust. The app’s rich information repository encompasses nutritional recommendations, product certifications, and other essential data. The app expedites access to critical product label information, including data pertaining to source, traceability, authenticity, and nutritional content. This information can be indispensable for individuals with food sensitivities or allergies, as well as those who seek to make informed choices while shopping. Accessing such data via the app can save users time and effort while also ensuring they select healthy options.

The user-friendly interface facilitates effortless access to this information, which is especially beneficial for individuals with dietary constraints or health conditions as it provides unambiguous guidance on essential nutrients. The ability to disseminate information in an accessible manner can be immensely beneficial for users, enabling them to make astute decisions regarding their dietary preferences. The emphasis on raising awareness regarding food products is commendable. This focus can enable individuals to make healthier choices and cultivate nutritional literacy. Furthermore, the app’s potential to reveal new markets for regional products and provide valuable data to users is noteworthy.

#### Barriers

Although two rounds of testing were conducted (Alpha and Beta) prior to the pilot, most of the feedback received pertained to challenges encountered with the usability and user experience of the Consumer App. It faces several barriers to its widespread adoption and usability. One of the main issues is the grouping of age, weight, and height, which does not account for the vast differences in body composition that can exist within each age range. Additionally, maintaining Bluetooth or IoT devices can be challenging, and some users may have difficulty using QR codes or beacons, especially older people who are not as familiar with technology. Inconsistencies and glitches within the app also make it difficult to use or understand, and some users may find it time-consuming to open the app to query information about products. All these factors contribute to a less than optimal user experience that can limit the app’s potential usefulness.

However, there are also several opportunities to improve the app and make it more accessible and valuable to users. For example, adding more details about each nutritional variable and allowing users to save their favourite meals could increase engagement and help users make more informed decisions about their food choices. Similarly, providing links to similar products and recipes could enhance the user experience and encourage continued use of the app. To improve accessibility for older users, the app could offer the option to zoom in on the page where users can scan QR codes, allowing people to access the app from different distances and positions. Finally, addressing the inconsistencies and glitches within the app and ensuring that it is user-friendly and intuitive would be crucial to overcoming the barriers that currently limit its adoption and usability.

#### Value of the consumer app solution

The main benefit deduced from the study is that the technological solution offers a user-friendly platform that enables users to efficiently track their food habits, plan a better diet, promote healthy diets, and raise awareness about the traceability of products. Moreover, the tool provides essential nutritional details about the products before purchase, enabling consumers to make informed decisions. The application further supports ensuring the traceability, certification, and nutritional value of produce or bulk products, which are not readily available through packaging labels.

There is a significant need for a technological solution that can offer effortless and immediate access to crucial information concerning food products, particularly regarding their nutritional value, traceability, and origin. The user interface should be intuitive, and the solution should foster healthy dietary habits and increase awareness about the significance of nutrition. It is also recommended that healthy-related tips and educational programs be incorporated to benefit all stakeholders. Ultimately, the solution should empower consumers to make more informed decisions regarding the food they purchase and consume, while also assisting them in planning their diets more efficiently.

## Discussion

The demographic characteristics of the study participants indicate that the Consumer App’s target audience is likely to be well-educated and technologically proficient individuals. However, the sample size is small and may not represent the broader population. Despite this, the high percentage of participants with advanced or excellent technological knowledge suggests that the app should focus on providing advanced features to cater to this user group.

The app’s acceptability and usability ratings suggest that while it may not be perfect, users generally find it acceptable and useful. Developers and designers should take note of the factors influencing user perception, such as ease of use and perceived usefulness, and work to improve these areas to increase adoption. The positive attitude of users towards the app indicates that there is a good chance of it being adopted, provided that it meets the users’ needs and expectations.

The analysis of the Consumer App’s usability and acceptability highlights the importance of conducting user testing to ensure that technological solutions meet users’ needs and expectations. While the app may be acceptable and usable to some users, there may be areas where it falls short for others. By gathering feedback from a diverse range of users and addressing their concerns, developers and designers can improve the usability and adoption potential of their technology. Overall, it is crucial to continue refining the Consumer App to make it more user-friendly and accessible to a wider range of users. It’s clear that there are several areas where the app can be improved to make it more user-friendly and accessible for a wider range of users.

To provide a way to save favourite food products with internal comments and links to similar products would allow users to easily access food products they have enjoyed in the past and discover new products that are similar to their favourite ones. Additionally, users could leave comments about the products they have saved, which could help others who are looking for recommendations.

To emphasize the importance of the certifications section would allow many users that may not be aware of the certifications that exist for different products, and highlighting this information could help users make more informed decisions about what they are consuming. In addition, providing a brief explanation of each certification and what it means would be helpful for users who are not familiar with them.

Adding recipes to the app could be valuable for users. This feature would allow users to search for recipes based on the products they have scanned, making it easier to plan meals and make healthier choices. The app could also suggest alternative ingredients or products to use in a recipe, based on the user’s dietary requirements or preferences. This feature could be especially useful for users who are new to healthy eating or have limited cooking experience.

It is important to address these issues to ensure that the app is accessible, user-friendly, and provides accurate and relevant information to its users. In addition to the recommendations listed above, providing an option to zoom in the page where you can scan the QR codes, splitting age, weight, and height to make it more accurate and personalized, and addressing inconsistencies and glitches in the app are also important areas to focus on.

## Conclusions

The feasibility study of the Med Food TTHubs Consumer App demonstrated its potential to enhance transparency, traceability, and consumer trust in the agri-food industry. The app’s comprehensive information on food products, from farm to fork, offers consumers valuable details regarding origin, authenticity, traceability, nutrition, and quality. The study revealed positive perceptions of the app’s usefulness and ease of use among participants, indicating its potential for successful adoption. However, certain barriers, such as usability issues and technological challenges, need to be addressed to improve the app’s overall user experience and maximize its potential benefits. By incorporating user feedback and making necessary improvements, the app can better meet user needs and facilitate informed decision-making regarding food choices.

The Consumer App holds significant value in empowering users to make informed decisions about their food choices, promoting healthy dietary habits, and instilling consumer trust. Its provision of comprehensive product information, including nutritional facts and certifications, facilitates convenient access to crucial data for individuals with dietary restrictions or allergies. The app’s user-friendly interface and emphasis on raising awareness about food products contribute to improved consumer experiences and nutritional literacy. Although certain barriers and challenges were identified, opportunities for enhancing the app’s accessibility and value were also highlighted. By addressing the identified barriers and incorporating additional features, such as detailed nutritional information and recipe suggestions, the app can further enhance user engagement and usefulness.

## Data Availability

The datasets generated and analysed during the current study are not publicly available due to ongoing research. Making the data public could jeopardize future research opportunities or competitive advantages for the authors or their affiliated institutions, but they are available from the corresponding author on reasonable request.

## Abbreviations

ENoLL: European Network of Living Labs
IoT: Internet of Things
ISCED: International Standard Classification of Education
LL: Living Lab
PRIMA: Partnership for Research and Innovation in the Mediterranean Area
SD: Standard Deviation
SMEs: Small and Medium-sized Enterprises
SUS: System Usability Scale
TAM: Technology Acceptance Model
TTHubs: Trace & Trust Hubs

## Code availability

The digital solution code is not available as open source; rather, it is proprietary and owned by Green Projects SA. Access to the code can be provided through appropriate channels and agreements with Green Projects SA.
